# Cytokinin Signaling in Mycobacterium tuberculosis

**DOI:** 10.1128/mBio.00989-18

**Published:** 2018-06-19

**Authors:** Marie I. Samanovic, Hao-Chi Hsu, Marcus B. Jones, Victoria Jones, Michael R. McNeil, Samuel H. Becker, Ashley T. Jordan, Miroslav Strnad, Changcheng Xu, Mary Jackson, Huilin Li, K. Heran Darwin

**Affiliations:** a Department of Microbiology, New York University School of Medicine, New York, New York, USA; b Van Andel Research Institute, Grand Rapids, Michigan, USA; c Human Longevity, Inc., San Diego, California, USA; d Mycobacteria Research Laboratories, Department of Microbiology, Immunology and Pathology, Colorado State University, Fort Collins, Colorado, USA; e Laboratory of Growth Regulators, Centre of the Region Haná for Biotechnological and Agricultural Research, Institute of Experimental Botany ASCR and Palacký University, Olomouc, Czech Republic; f Biology Department, Brookhaven National Laboratory, Upton, New York, USA; Seattle Biomedical Research Institute; University of Washington

**Keywords:** Mycobacterium tuberculosis, acid-fast staining, cytokinin, regulation, signaling

## Abstract

It was recently reported that the human-exclusive pathogen Mycobacterium tuberculosis secretes cytokinins, which had only been known as plant hormones. While cytokinins are well-established, adenine-based signaling molecules in plants, they have never been shown to participate in signal transduction in other kingdoms of life. M. tuberculosis is not known to interact with plants. Therefore, we tested the hypothesis that cytokinins trigger transcriptional changes within this bacterial species. Here, we show cytokinins induced the strong expression of the M. tuberculosis gene Rv0077c. We found that Rv0077c expression is repressed by a TetR-like transcriptional repressor, Rv0078. Strikingly, cytokinin-induced expression of Rv0077c resulted in a loss of acid-fast staining of M. tuberculosis. While acid-fast staining is thought to be associated with changes in the bacterial cell envelope and virulence, Rv0077c-induced loss of acid-fastness did not affect antibiotic susceptibility or attenuate bacterial growth in mice, consistent with an unaltered mycolic acid profile of Rv0077c-expressing cells. Collectively, these findings show cytokinins signal transcriptional changes that can affect M. tuberculosis acid-fastness and that cytokinin signaling is no longer limited to the kingdom Plantae.

## IMPORTANCE

Cytokinins have only previously been known as plant hormones. The discovery that they can be used as signaling molecules outside of plants broadens the repertoire of small molecules that can potentially affect gene expression in all domains of life.

## INTRODUCTION

Mycobacterium tuberculosis is the causative agent of tuberculosis, one of the world’s leading causes of mortality (1). For this reason, researchers are eager to identify pathways that could be targeted for the development of new therapeutics to treat this devastating disease. Among the current prioritized targets is the mycobacterial proteasome. M. tuberculosis strains with defects in proteasome-dependent degradation are highly attenuated in mice, partly because they are sensitive to nitric oxide (NO) (2–7). The NO-sensitive phenotype of mutants defective for proteasomal degradation has been attributed to a failure to degrade an enzyme called Log (Lonely guy), a homologue of a plant enzyme involved in the synthesis of a family of *N*^*6*^-substituted adenine-based molecules called cytokinins (CKs) (8). The accumulation of Log in M. tuberculosis results in a buildup of cytokinins, which can break down into aldehydes that effectively sensitize mycobacteria to NO (8).

While we determined that a lack of proteasome-dependent degradation results in cytokinin accumulation, we were left with more questions, namely, what is the function of cytokinin production by M. tuberculosis? In plants, cytokinins are hormones that regulate growth and development (9). In addition, bacterial plant pathogens and symbionts use cytokinins to facilitate the parasitism of their plant hosts (10). Outside of the laboratory, M. tuberculosis exclusively infects humans and is not known to have an environmental reservoir; therefore, it is unlikely M. tuberculosis secretes cytokinins to modulate plant development. Instead, we hypothesized that M. tuberculosis, like plants, uses cytokinins to signal intraspecies transcriptional changes to its benefit. Here, we show that cytokinins induce the transcription of a gene of unknown function. Moreover, we identified and characterized a TetR-like regulator that represses the expression of this gene. While we have not yet identified an *in vivo* phenotype associated with this cytokinin-inducible gene, we found that its expression altered the cell envelope of M. tuberculosis, changing its staining properties. Collectively, these studies provide a foundation to characterize cytokinin signaling in M. tuberculosis and other cytokinin-producing bacterial species.

## RESULTS

### Cytokinins induce the specific and high expression of Rv0077c in M. tuberculosis*.*

**FIG 1:**
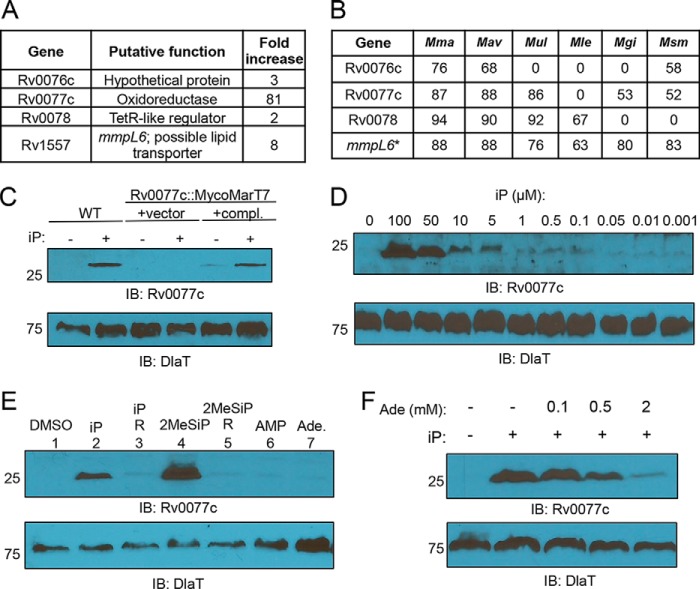
Cytokinins induce the expression of Rv0077c in M. tuberculosis. (A) Genes significantly regulated by the presence of 100 µM iP for 5 h as analyzed by RNA-Seq. (B) Percentage of identity between M. tuberculosis H37Rv proteins and proteins of targeted mycobacterial genomes, including M. marinum (Mma), M. avium (Mav), M. ulcerans (Mul), M. leprae (Mle), M. gilvum (Mgi), and M. smegmatis (Msm). An asterisk indicates *mmpL6* encodes a truncated protein in H37Rv, unlike in the other mycobacterial species in the table. (C) Immunoblot for Rv0077c in total cell lysates of WT M. tuberculosis. “compl.” indicates complemented. iP was used at a final concentration of 100 µM when added. (D) Dose-dependent production of Rv0077c protein. Bacteria were incubated with cytokinin at the indicated concentrations for 24 h. (E) Only cytokinins, and not closely related molecules, induce the production of Rv0077c. Each compound was added to a final concentration of 100 µM. “R” indicates the riboside form of the preceding indicated cytokinin. (F) Adenine inhibits the induction of Rv0077c by 100 µM iP. For all panels, we added an equal volume of DMSO to samples where iP was not added. For all immunoblots (IB), we stripped the membranes and incubated them with antibodies to dihydrolipoamide acyltransferase (DlaT) to confirm equal loading of total lysates. Molecular weight standards are indicated to the left of the blots and are in kilodaltons (kDa). Ade, adenine.

To test if a cytokinin (CK) could induce gene expression in M. tuberculosis, we grew wild-type (WT) M. tuberculosis H37Rv to mid-logarithmic phase and incubated the bacteria for 5 h with *N*^*6*^-(Δ^2^-isopentenyl)adenine (iP), one of the most abundantly produced cytokinins in M. tuberculosis, which is also commercially available (8) (see Materials and Methods and Tables 1 and 2 for strains, plasmids, primers, and probes). Using high-throughput RNA sequencing (RNA-Seq), we discovered the expression of four genes, Rv0076c, Rv0077c, Rv0078, and *mmpL6*, was significantly induced upon iP treatment compared to treatment with the vehicle control (dimethyl sulfoxide [DMSO]) (Fig. 1A; see Table S1 in the supplemental material). Rv0077c is conserved among many mycobacterial species, while Rv0076c and Rv0078 are present only in several mycobacterial genomes (Fig. 1B) (11). Notably, Mycobacterium smegmatis, a distant, nonpathogenic relative of M. tuberculosis, has a weak homologue of Rv0077c and no conspicuous Rv0078 homologue (Fig. 1B). *mmpL6* is one of 13 *mmpL* (mycobacterial membrane protein large) genes in M. tuberculosis. In strain H37Rv, *mmpL6* is predicted to encode a 42-kDa protein with five transmembrane domains and is truncated compared to the same gene in ancestral tuberculosis strains (12). Thus, it is unclear if *mmpL6* encodes a functional protein in strain H37Rv.

10.1128/mBio.00989-18.6 TABLE S1  RNASeq analysis of M. tuberculosis strain H37Rv treated with either iP or DMSO (vehicle control) for 5 h at 37°C shows changes in gene expression. RNA was collected and analyzed as described in Materials and Methods. Biological triplicate samples were analyzed. The list is presented from highest increased changes (highlighted in red) to most reduced gene expression in the iP-treated versus DMSO-treated cultures (in green). Download TABLE S1, XLSX file, 0.9 MB. Copyright © 2018 Samanovic et al. 2018 Samanovic et al. This content is distributed under the terms of the Creative Commons Attribution 4.0 International license.

**TABLE 1:** Bacterial strains and plasmids used in this study

Strain or plasmid	Relevant genotype	Source or reference
Strains		
M. tuberculosis		
H37Rv	Wild type (WT)	ATCC 25618
MHD18	Hyg^r^ WT with pMV306	3
MHD761	Strep^r^ WT with pMV306.strep	8
MHD794	Kan^r^ WT with pMV306.kan	44
MHD1033	Kan^r^ Rv0077c::MycoMarT7 with transposon insertion between codons 64 and 65	This work
MHD1077	Kan^r^ Strep^r^ MHD1033 with pMV306.strep-Rv0077c	This work
MHD1086	Kan^r^ Strep^r^ MHD1033 with pMV306.strep	This work
MHD1293	Hyg^r^ ΔRv0078::*hyg*	This work
MHD1315	Hyg^r^ Kan^r^ ΔRv0078::*hyg* with pMV306.kan	This work
MHD1316	Hyg^r^ Kan^r^ ΔRv0078::*hyg* with pMV306.kan-Rv0078_W100R_	This work
MHD1318	Hyg^r^ Kan^r^ ΔRv0078::*hyg* with pMV306.kan-Rv0078	This work
E. coli		
ER2566	F^−^ λ^−^ *fhuA2* [lon] *ompT lacZ*::T*7 geneI gal sulA11* Δ(*mcrC-mrr*)*114*::IS*10*R(*mcr-73*::miniTn*10*) 2R(*zgb-210*::Tn*10*)*1* (Tet^s^) *endA1* [dcm]	45
DH5α	F^−^ φ80d*lacZ*ΔM15 Δ(*lacZYA-argF*)*U169 deoR recA1 endA1 hsdR17* (r_K_^−^ m_K_^+^) *phoA supE44* λ *thi-1 gyrA96 relA1*	Gibco, BRL

Plasmids		
pET24b(+)	Kan^r^ plasmid for production of C-terminal His_6_ epitope-tagged protein	Novagen
pMV306.kan	Kan^r^ plasmid that integrates at *attB* site on *M. tuberculosis* chromosome	46
pMV306	Hyg^r^ plasmid that integrates at *attB* site on *M. tuberculosis* chromosome	46
pMV306.strep	Strep^r^ plasmid that integrates at *attB* site on *M. tuberculosis* chromosome	Gift from John Mckinney
pYUB854	Hyg^r^ allelic exchange vector	34
pMV306.strep-Rv0077c	Strep^r^ pMV306.strep plasmid with Rv0077c and 200 bp upstream of start codon of Rv0077c	This work
pMV306.kan-Rv0078	Kan^r^ pMV306.kan plasmid with Rv0078 and 200 bp upstream of start codon of Rv0078	This work
pMV306.kan-Rv0078_W100R_	Kan^r^ pMV306.kan-Rv0078 plasmid with Trp100 changed to Arg	This work
pET24b(+)-Rv0077cHis_6_	Kan^r^; for production of Rv0077c-His_6_ in E. coli	This work
pET24b(+)-Rv0078His_6_	Kan^r^ plasmid for production of Rv0078-His_6_ in E. coli	This work

**TABLE 2:** Sequences of the primers and probes used in this study

Primer or probe	Sequence^a^	Purpose
Primers		
F2Rv0077cNde1	CAGTCAT**ATG**TCGACGATCGACATTAGTGCCG	For cloning into pET24b(+)
R20077cHindIII	GTCT**AAGCTT**CGTGCGCACCGCGACCGTCGACAACTG	For cloning into pET24b(+)
F2Rv0078Nde1	TAGTCATATGGAAATCAAGAGACGCACCCAGGAG	For cloning into pET24b(+)
R20078HindIII	AATG**AAGCTT**GCCGTTAAGCATCCCGTCGATGAG	For cloning into pET24b(+)
F3Rv00777cFISH	TGACCCCCGCCTCGGTCGCGATTTCCG	For mutant mining of Rv0077c::MycoMarT7
F5Rv0077cNhe1	TATCGCTAGCTGACCCCCGCCTCGGTCGCGATTTCCG	Rv0077c complementation in M. tuberculosis in pMV306.strep
R4Rv0077cHindIII	AGGTAAGCTTCTACGTGCGCACCGCGACCGTCGACAACTG	Rv0076c-Rv0077c complementation in M. tuberculosis in pMV306.strep
R4Rv0076cHindIII	TAGTAAGCTTTCACAACGCTGCGGCGTGTTGGGTC	Rv0076c-Rv0077c complementation in M. tuberculosis in pMV306.strep
R2Rv00785Race	CCAGGCATCGACCGCTGCCCGG	5′ RACE of Rv0078
R2Rv0077c5Race	GCCGGTGTCGTTGCCGACCAGCA	5′ RACE of Rv0077c
R1Rv0077c5Race	GCGACGAGCTGGGTGACGAC	5′ RACE of Rv0077c
R1Rv00785Race	GGATCACCAGATACCTCGAG	5′ RACE of Rv0078
F5Rv0078Nhe1	TATCGCTAGCGACCGGCGAGTCGCTCACTGACCCGTCGCCATAG	Rv0078 complementation in pMV306.kan
R5Rv0078HindIII	CGGTAAGCTTCTAGCCGTTAAGCATCCCGTCGATG	Rv0078 complementation in pMV306.kan

EMSA probes		
+74/+32	AATGAATAGTTCCGGCACTAATGTCGATCGTCGACATGGATGCCCAGACCAGGGCAC	
+8/−25	AGCAGACTGCCGGTAACTTACCAACAGATTGTACCAGACCAGGGCAC	
+19/−14 (WT)	GTACATTTACAAGCAGACTGCCGGTAACTTACCCCAGACCAGGGCAC	
+19/−14 NS11	GTACATGATAAAGCAGACTGCCTAGTGATAGTGCCAGACCAGGGCAC	
+19/−14 NS21	GTACATGATAGTAGTAGTGATGATGATCTTACCCCAGACCAGGGCAC	
+19/−14 NS27	GTACATGATAGTAGTAGTGATGATGATATAGTGCCAGACCAGGGCAC	
14merScafold5IRDye700	5′-5IRD700-GTGCCCTGGTCTGG	

^a^ In the primer sequences, changes to the normal sequence are in boldface. In the probe sequences, the 14-nucleotide oligomers are underlined.

Rv0077c was by far the most strongly induced gene in M. tuberculosis upon iP treatment; therefore, we chose it for follow-up studies. We raised polyclonal antibodies to recombinant Rv0077c protein and showed that protein levels were increased in M. tuberculosis treated with iP for 24 h (Fig. 1C, first two lanes). Rv0077c was barely detectable in cell lysates of bacteria that had not been incubated with iP and was undetectable in a strain with a transposon insertion mutation in Rv0077c (Fig. 1C, center two lanes). Rv0077c protein was restored to WT levels in the mutant upon complementation with an integrative plasmid carrying Rv0077c expressed from its native promoter (Fig. 1C, last two lanes). We also found a dose-dependent induction of Rv0077c production using iP concentrations from 1 nM to 100 µM (Fig. 1D).

We next synthesized and tested if the most abundantly produced cytokinin in M. tuberculosis, 2-methylthio-iP (2MeSiP) (8), could also induce Rv0077c production. 2MeSiP strongly induced Rv0077c production (Fig. 1E, lane 4). Importantly, we did not observe induction of Rv0077c when we incubated the bacteria with the appropriate cytokinin riboside (R) precursors iPR or 2MeSiPR (Fig. 1E, lanes 3 and 5). Similarly, AMP or the closely related molecule adenine could not induce Rv0077c expression (Fig. 1E, lanes 6 and 7). We hypothesized while adenine could not induce Rv0077c expression, at high enough concentrations, it could possibly inhibit Rv0077c induction by competing with cytokinin for access to a transporter or receptor. Indeed, adenine reduced the induction of Rv0077c by iP in a dose-dependent manner (Fig. 1F).

### Identification of an operator for the TetR-like transcriptional repressor Rv0078.

**FIG 2:**
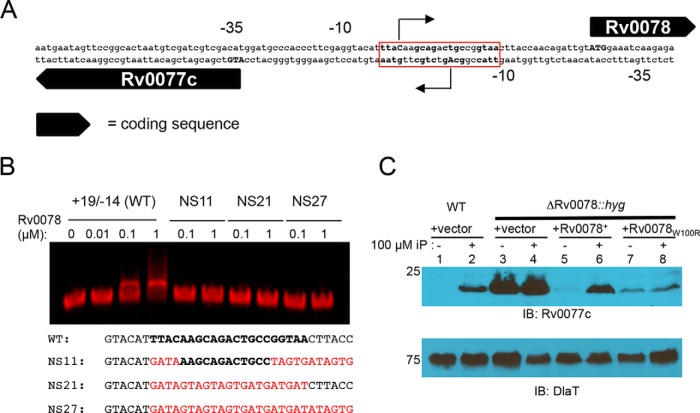
Rv0078 represses the expression of Rv0077c. (A) The putative transcriptional start sites (+1) of Rv0077c and Rv0078 as determined by 5′ RACE and represented as bent arrows. The predicted start codons are in capital boldface letters. (B) EMSA analysis identifies a putative repressor binding site. Probe sequence +19/−14 refers to positions relative to Rv0077c position +1. In boldface is the presumed binding site. Mutated residues are in red. Not shown at the end of each probe is a sequence for annealing to a fluorescent tag (Table S1). Rv0078 was purified under native conditions from E. coli. (C) Deletion and disruption of Rv0078 result in the constitutive expression of Rv0077c. Total cell lysates were prepared and separated on a 10% SDS-PAGE gel. IB, immunoblot. The membrane was stripped and then incubated with antibodies to DlaT to confirm equal loading of samples. Molecular weight standards are indicated to the left of the blots and are in kilodaltons (kDa).

Rv0077c is divergently expressed from Rv0078; the proposed translational start codons for these genes are separated by 61 bp. Based on its cytokinin-dependent expression and its proximity to Rv0077c, we hypothesized that Rv0078 encodes a repressor of both genes. We identified the promoter regions for each gene by performing rapid amplification of 5′ cDNA ends (5′ RACE) analysis of Rv0077c and Rv0078 and determined the likely start of transcription for one gene was within the 5′ untranslated region of the other gene (Fig. 2A). We sought to identify an operator sequence by using an electrophoretic mobility shift assay (EMSA) (Fig. 2B). We narrowed down a putative Rv0078 binding site to a region overlapping the proposed starts of transcription (+1) of both Rv0077c and Rv0078 (Fig. 2A, red box). TetR-like regulators generally bind to inverted repeat sequences; we identified a 21-bp sequence within positions −14/+19 (relative to position +1) of Rv0077c containing two 10-bp inverted repeat sequences. Mutagenesis of the probe in the repeat sequences disrupted Rv0078 binding to the DNA (Fig. 2B). Importantly, the binding sequence overlaps the putative transcriptional start sites of both genes, suggesting that expression of both genes is repressed by Rv0078. Notably, the addition of iP did not result in the release of Rv0078 from the DNA probe (Fig. 2B; see Fig. S1 in the supplemental material). Therefore, cytokinins do not appear to directly bind to Rv0078 to induce gene expression.

10.1128/mBio.00989-18.1 FIG S1  The cytokinin iP cannot dissociate Rv0078 from DNA. A 6% TBE gel was imaged using a Li-Cor Odyssey imager. Ten millimoles MgCl_2_ improved DNA binding. Download FIG S1, PDF file, 0.1 MB. Copyright © 2018 Samanovic et al. 2018 Samanovic et al. This content is distributed under the terms of the Creative Commons Attribution 4.0 International license.

Based on the 5′ RACE analysis, we were able to delete and replace most of the Rv0078 gene with the hygromycin resistance gene (*hyg*) without disrupting the promoter of Rv0077c in M. tuberculosis H37Rv. A ΔRv0078::*hyg* strain displayed constitutively high expression of Rv0077c irrespective of the presence of cytokinin, supporting a model in which Rv0078 directly represses Rv0077c expression (Fig. 2C). A single copy of Rv0078 expressed from its native promoter restored iP-regulated control of Rv0077c in this strain (Fig. 2C, lanes 5 and 6). Interestingly, in the process of making the complementation plasmid, we acquired a random mutation (likely generated during PCR) in Rv0078 that changed a tryptophan to arginine (W100R); this allele was unable to restore cytokinin-dependent regulation of Rv0077c to the ΔRv0078::*hyg* strain (Fig. 2C, lanes 7 and 8).

### Two Rv0078 dimers bind to one operator.

**FIG 3:**
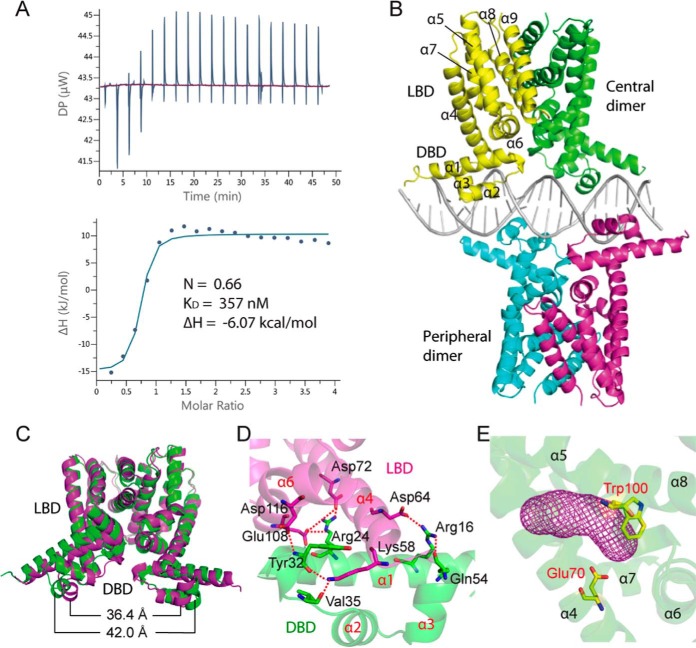
Crystal structure of Rv0078 in complex with DNA. (A) ITC of Rv0078 binding to the +13/−8 DNA probe. The binding stoichiometry, ΔH, and *K*_*D*_ are marked. (B) Overall structure of Rv0078-DNA complex in cartoon view. Two Rv0078 dimers (“central” and “peripheral”) bind to one DNA molecule. (C) The distance between two DNA-binding domains decreases by ~6 Å when bound to DNA. The DNA-free Rv0078 is in green, and the DNA-bound Rv0078 is in magenta. (D) Interactions between the LBD (magenta) and DBD (green) in a monomer. (E) The ligand binding pocket (magenta mesh) of Rv0078 is enclosed by a four-helix bundle (helices α5 to α8).

To gain an understanding of how Rv0078 represses gene expression, we solved the crystal structure of Rv0078 to 1.85 Å by a single-wavelength anomalous dispersion method. As previously reported, Rv0078 forms dimers resembling other TetR-like proteins (13, 14). Unlike the canonical TetR binding site, *tetO*, which is 15 bp long, the Rv0078 binding site is 21 bp long, suggesting Rv0078 binds to DNA differently than TetR. We performed an isothermal titration calorimetry (ITC) experiment and determined that the apparent *K*_*D*_ (dissociation constant) of Rv0078 for operator DNA was 357 nM (Fig. 3A). The estimated stoichiometry of DNA duplex to Rv0078 dimer was 0.66, suggesting that two Rv0078 dimers bind to one operator sequence.

We next cocrystallized Rv0078 with a 23-bp DNA fragment that was extended 1 bp at each end of the 21-bp operator sequence identified in Fig. 2 (positions −8/+13 [relative to +1] of Rv0077c) and solved the structure to a resolution of 3.0 Å (Table 3). As predicted by the ITC experiments, we observed two Rv0078 dimers bound to a DNA duplex where each dimer bound on opposite sides of the DNA (Fig. 3B). The top or “central” dimer bound the DNA palindrome symmetrically, while the bottom or “peripheral” dimer bound to DNA off-center, staggered from the central dimer binding site by 7 bp. Thus, the longer-than-canonical (i.e., *tetO*) binding site is required for accommodation of two Rv0078 dimers, unlike TetR, which binds to *tetO* as a single dimer.

**TABLE 3:** Data collection and refinement statistics for Rv0078 with and without DNA

Parameter	Value(s) for^a^:		
	Rv0078	Rv0078 DNA	SeMet Rv0078
Data collection statistics			
Wavelength	0.97931	1.12713	0.97931
Space group	C121	P12_1_1	C121
Unit cell dimensions			
*a*, *b*, *c* (Å)	224.91, 67.07, 56.02	82.04, 82.04, 186.66	219.26, 68.04, 57.49
α, β, γ (°)	90, 99.19, 90	90, 90.07, 90	90, 97.06, 90
Resolution (Å)	111.01–1.85 (1.95–1.85)	28.75–3.0 (3.16–3.00)	108.8–2.7 (2.85–2.7)
*R*_merge_ (%)	5.1 (42.1)	20.4 (105)	12.6 (47.3)
*I*/σ*I*	16.97 (2.7)	5.5 (1.8)	12.7 (4.6)
Total reflections	252,128	296,694	175,154
Completeness (%)	96.5 (95.1)	94.8 (100)	99.5 (100)
Redundancy	3.7 (3.5)	6.4 (5.8)	3.9 (3.9)
Refinement statistics			
Resolution (Å)	36.38–1.85	28.5–3.0	
No. of reflections	67,720	46,083	
*R*_work_/*R*_free_ (%)	0.2174/0.2469	0.2019/0.2469	
No. of:			
Nonhydrogen atoms	6,037	13,639	
Macromolecules	5,937	13,639	
Ligand	ND	ND	
Water	100		
*B*-factors (Å^2^)	30.68	69.15	
Macromolecules	30.78	69.15	
Water	24.65		
RMSDs			
Bond lengths (Å)	0.007	0.10	
Bond angles (°)	0.83	1.21	
Ramachandran statistics (%)			
Favored	99.35	97.53	
Allowed	0.65	2.47	
Outliers	0	0	

^a^ Values in parentheses are for the highest-resolution shell. ND, not determined.

Rv0078 has an N-terminal DNA-binding domain (DBD) and a C-terminal ligand-binding domain (LBD) (Fig. 3B to D). DNA binding induced significant conformational changes across the Rv0078 dimer structure, with a root mean square deviation (RMSD) of 2.13 Å compared to the DNA-free dimer. In particular, the two α3 helices move toward each other by ~6 Å, in order to reduce their distance to 36.4 Å and fit in the DNA major grooves (Fig. 3C). In the LBD, the ligand entry between helices α4 and α5 is open and the ligand-binding pocket is empty. It appears that changes initiated in the DBD regions upon binding to the DNA are transmitted to the LBD via the DBD-LBD interface, which involves extensive interactions, including two salt bridges and six H-bonds (Fig. 3D).

The ligand-binding pocket of Rv0078 is largely hydrophobic. Within this pocket, we observed an elongated density resembling a long aliphatic chain of a fatty acid (see Fig. S2 in the supplemental material). Gas chromatography-mass spectrometry (GC-MS) of the compounds extracted from Rv0078 purified from Escherichia coli revealed fatty acids commonly found in this organism, and a palmitate molecule fit well with the electron density (Fig. S2A to E). A fatty acid carboxylate formed a hydrogen bond with Rv0078 Glu-70 (Fig. 3E and Fig. S2C), and the long alkyl chain had numerous hydrophobic interactions within the extended ligand pocket. When we purified Rv0078 under denaturing conditions and refolded the protein to remove the lipid, we observed the same EMSA results as we observed with protein purified under native conditions (data not shown), suggesting this fatty acid is unlikely to be the native Rv0078 ligand; however, these data may suggest that the native ligand is fatty acid-like. Interestingly, we found that Trp100 faces the ligand-binding pocket (Fig. 3E), which suggests Trp100 interacts with the natural ligand. This hypothesis is supported by our data showing an Rv0078_W100R_ mutant was unable to complement the Rv0078 deletion mutant strain (Fig. 2C, lane 8).

10.1128/mBio.00989-18.2 FIG S2  Rv0078 purified from E. coli binds fatty acids. (A) GC-MS of fatty acids extracted from E. coli*-*expressed and purified Rv0078. Each identified fatty acid peak is labeled by the total number of carbon followed by the number of unsaturated C-C bonds. IS, internal standard; UN, unknown peak. (B) Quantification of the relative amount of fatty acids bound to Rv0078. (C) A palmitate fits the 2*F*_o_ − *F*_c_ density map in the ligand binding pocket. The density is contoured at 1σ in blue mesh. (D) The structure of Rv0078 bound to a fatty acid. The protein is shown in a rainbow cartoon and the ligand in spheres. Only one monomer of the dimer is shown. (E) Except for an H-bond with Glu-70, the palmitate is surrounded by hydrophobic residues of the ligand-binding pocket. This panel was generated with the program Ligplot+ (R. A. Laskowski and M. B. Swindells, J Chem Inform Model, 51:2778–2786, 2011, https://doi.org/10.1021/ci200227u). Download FIG S2, PDF file, 1 MB. Copyright © 2018 Samanovic et al. 2018 Samanovic et al. This content is distributed under the terms of the Creative Commons Attribution 4.0 International license.

**FIG 4:**
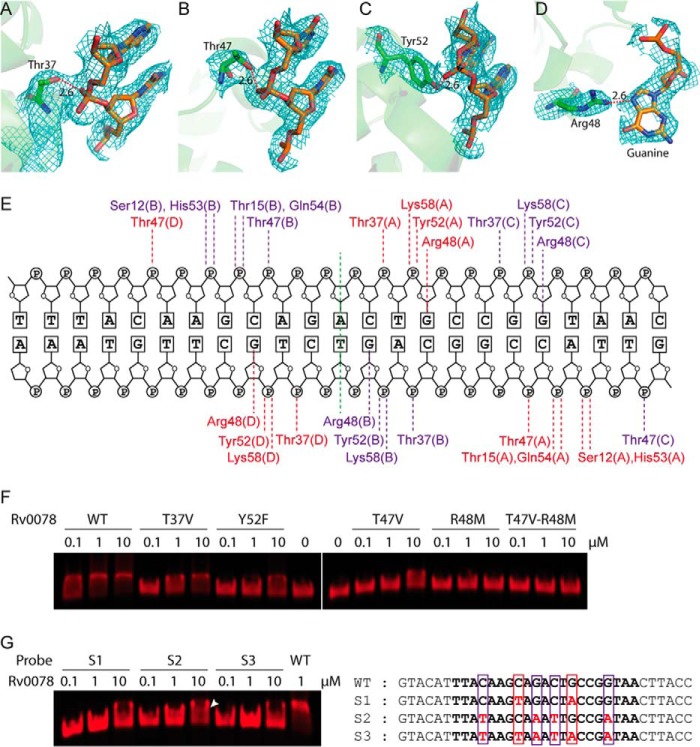
Rv0078-DNA interactions determined by X-ray crystallography. The hydroxyl groups of Thr37 (A), Thr47 (B), and Tyr52 (C) interact with the backbone phosphate with a distance of 2.6 Å. (D) Arg48 interacts with guanidine with a distance of 2.6 Å. The 2*F*_o_ − *F*_c_ maps are contoured at the 1σ level. (E) A schematic representation of Rv0078-DNA contacts. Residues of the central dimer are labeled in red, and residues of the peripheral dimer are in purple. (F) EMSA using WT DNA probe and Rv0078 with mutations in Thr37, Thr47, Arg48, or Tyr52. (G, left panel) EMSA of Rv0078 with DNA probes with G-to-A substitutions in the central dimer binding region (S1), peripheral dimer binding region (S2), or in both DNA regions (S3). The white arrowhead marks the partial shift with the S2 probe at high protein concentration. (G, right panel) Sequences of the four DNA probes used in EMSAs. The nucleotides contacting the central dimer are boxed in red, and those contacting the peripheral dimer are boxed in purple.

In addition to characterizing the LBD, we identified 9 amino acids that were important for interacting with DNA (Fig. 4A to E). Specifically, the hydroxyl groups of Thr37, Thr47, and Tyr52 interacted strongly with DNA phosphates at a distance of 2.6 Å. Arg48 is the only residue that interacted with DNA by recognizing a guanine base at a distance of 2.6 Å. To further examine the importance of these residues, we introduced single amino acid substitutions (T37V, T47V, R48M, and Y52F) or double mutations (T47V and R48M) into Rv0078 and performed EMSAs. While all of the mutant proteins were soluble and behaved like the WT protein in solution, Rv0078_R48M_ did not bind to DNA, and the other mutant proteins bound to DNA with reduced affinities (Fig. 4F).

To examine the DNA sequence specificity of Rv0078, we synthesized three EMSA probes by changing the Rv0078 Arg48-interacting guanines to adenines (G-to-A) in the central dimer binding region (probe S1), the peripheral dimer-binding region (probe S2), or both dimer-binding regions (probe S3). These G-to-A substitutions either abolished or compromised Rv0078 binding to DNA, affirming the critical role of guanines in the binding site (Fig. 4G). Notably, substitutions in probe S1 entirely abolished Rv0078 binding, while substitutions in S2 retained a partial gel shift at a high concentration (white arrowhead in Fig. 4G). This observation suggests that the binding of the two Rv0078 dimers is cooperative, with the central dimer likely the first one to bind to DNA.

### Constitutive expression of Rv0077c does not affect antibiotic susceptibility or virulence in mice.

During our ongoing studies, a report was published on the identification of a small molecule of the spiroisoxazoline family, SMARt-420, which strongly induces the expression of the Rv0077c orthologue *bcg_0108c* in M. bovis bacillus Calmette-Guerin (15). Using X-ray crystallography and surface plasmon resonance techniques, the authors of this study found SMARt-420 binds to Rv0078 to derepress binding from the Rv0077c promoter (14, 15). SMARt-420 was identified in a search for compounds that boost the efficacy of the second-line tuberculosis drug ethionamide (ETH). ETH is a prodrug that is activated by the mono-oxygenase EthA, which transforms ETH into highly reactive intermediates. Activated ETH and NAD form a stable adduct that binds to and inhibits InhA, an essential enzyme needed for mycolic acid synthesis in mycobacteria (16, 17). While spontaneous inactivating mutations in *ethA* result in resistance to ETH, it was proposed that the induction of Rv0077c expression could bypass the need for EthA and transform ETH into its toxic form (15). Based on this study, we predicted that an Rv0078 mutant of M. tuberculosis, which expresses high levels of Rv0077c, should be hypersensitive to ETH compared to the parental strain H37Rv. However, we observed either little to no significant change in the 50% inhibitory concentration (IC_50_) of ETH between the WT and ΔRv0078::*hyg* strains (Table 4) or in WT M. tuberculosis H37Rv treated with or without 100 µM iP (IC_50_, 1.4). We also tested if the constitutive expression of Rv0077c changed the susceptibility of M. tuberculosis to other antibiotics, including two cell wall synthesis inhibitors. We observed no differences in the IC_50_s of these antibiotics between the WT and ΔRv0078::*hyg* strains (Table 4).

**TABLE 4:** Antibiotic IC_50_s of the WT and Rv0078 strains

Drug	IC_50_ for:	
	Parental strain	ΔRv0078::*hyg* mutant
Ethionamide	1.0–2.3 µg/ml	0.8–1.3 µg/ml
Ciprofloxacin	0.1–0.2 µg/ml	0.1–0.2 µg/ml
Ethambutol	0.6–2 µg/ml	0.6–2 µg/ml
Isoniazid	32–44 ng/ml	29–44 ng/ml
Meropenem^a^	1 µg/ml	1 µg/ml
Norfloxacin	2 µg/ml	2 µg/ml
Rifampin	32–38 ng/ml	32–38 ng/ml
Streptomycin	0.3–0.6 µg/ml	0.3–0.4 µg/ml
Vancomycin^a^	3–10 µg/ml	6–10 µg/ml

^a^ Supplemented with 5 µg/ml potassium clavalunate. See Materials and Methods for details.

**FIG 5:**
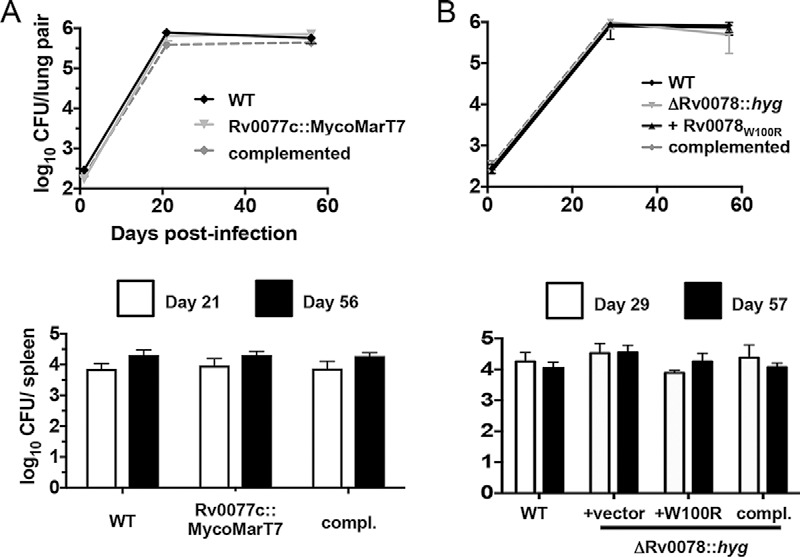
Loss of Rv0077c or Rv0078 does not attenuate bacterial survival in mice. (A) Bacterial CFU after infection of C57BL/6J mice with the WT, Rv0077c mutant, and complemented strains. (B) Bacterial CFU after infection of C57BL/6J mice with the WT, Rv0078 mutant, and complemented strains. For both panels, data in each are from a single experiment that is representative of two independent experiments. Error bars indicate the standard error of the mean.

We also tested if either the Rv0077c or Rv0078 mutant had growth defects *in vivo* compared to the WT H37Rv strain. We infected C57BL/6J mice by a low-dose aerosol route with the parental, mutant, and complemented mutant strains, as well as with the Rv0078 mutant transformed with the Rv0078_W100R_ allele. None of the strains revealed a difference in growth or survival compared to WT M. tuberculosis in mice, as determined by the recovery of CFU from the lungs and spleens (Fig. 5; see Fig. S3 in the supplemental material).

10.1128/mBio.00989-18.3 FIG S3  An Rv0078 deletion disruption mutant, which overexpresses Rv0077c, has no long-term virulence defect in C57BL6/J mice. For each experiment, 16 mice were infected with 200 to 400 CFU of each strain (64 mice/experiment) by the aerosol route of infection. See Materials and Methods for details. MHD794 is the WT with empty vector, MHD1315 is the ΔRv0078::*hyg* mutant with empty vector, MHD1316 is the ΔRv0078::*hyg* mutant with Rv0078_W100R_, and MHD1318 is the ΔRv0078::*hyg* mutant complemented with WT Rv0078. Error bars indicate the standard error of the mean. Download FIG S3, PDF file, 0.2 MB. Copyright © 2018 Samanovic et al. 2018 Samanovic et al. This content is distributed under the terms of the Creative Commons Attribution 4.0 International license.

### Expression of Rv0077c alters acid-fast staining of M. tuberculosis*.*

**FIG 6:**
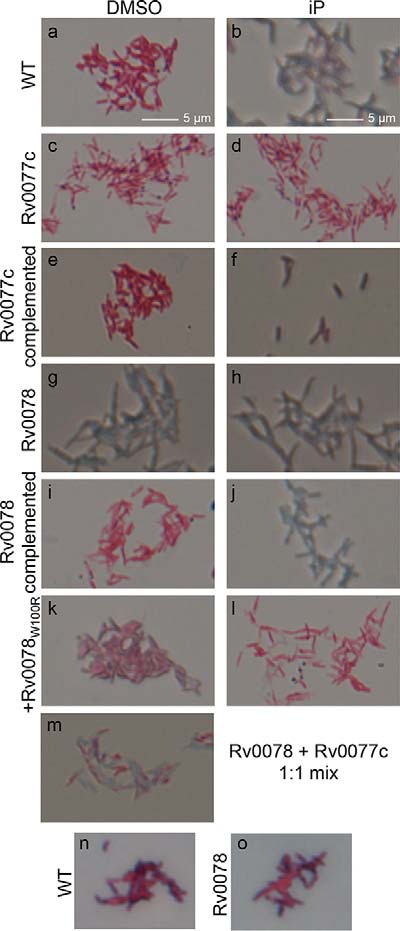
Induction of Rv0077c expression results in loss of acid-fast staining. M. tuberculosis strains were examined by Ziehl-Neelsen staining. The magnification is 63-fold. Scale bars are applicable to images in panels a to m. See text for details. “Rv0077c” indicates the Rv0077c::MycoMarT7 strain and “Rv0078” indicates the ΔRv0078::*hyg* strain.

To gain insight into the function of Rv0077c, we performed metabolomic analysis of strains expressing Rv0077c to potentially determine how its presence alters bacterial physiology. We prepared total cell lysates of WT and Rv0077c mutant strains treated with or without 100 µM iP for 24 h (see Materials and Methods). From a total of 337 detectable metabolites, we observed a significant change in 24 molecules after the addition of iP to WT M. tuberculosis. Seventeen metabolites showed a consistent difference between samples in which iP was added, and these changes disappeared in an Rv0077c-disrupted strain, suggesting the changes were specifically due to the presence of Rv0077c (Table S2, highlighted in yellow). We observed an increased abundance of several phospholipids and a decrease in a major precursor of peptidoglycan, *N*-acetylglucosamine-1-phosphate (GlcNAc1P). We therefore hypothesized that Rv0077c modified one or more components of the cell envelope. Microscopic examination of Ziehl-Neelsen (ZN)-stained WT M. tuberculosis treated with iP showed a loss of acid-fast staining (Fig. 6a versus b), and this phenotype depended on the presence of Rv0077c (Fig. 6c versus d). Complementation of the mutation with Rv0077c alone restored the iP-induced loss of acid-fast staining (Fig. 6e and f). Deletion of Rv0078 resulted in a constitutive loss of staining, irrespective of the presence of iP (Fig. 6g and h). Complementation of the Rv0078 deletion with the WT gene restored iP control of loss of acid-fast staining (Fig. 6i and j), but complementation with Rv0078_W100R_, which could not fully derepress Rv0077c expression in the presence of iP (Fig. 2C), could not restore iP-induced loss of acid-fast staining (Fig. 6k and l). Mixing and simultaneous staining of the Rv0077c and Rv0078 mutants further showed the staining differences were not a result of a technical artifact (Fig. 6m). We also tested whether or not a carbolfuchsin (the primary stain in ZN staining) could interact at all with bacteria expressing Rv0077c and found that both the Rv0078 mutant and the parental strain stained equally well (Fig. 6n and o). This result suggested Rv0077c-expressing bacteria could bind to stain but not retain it after acid washing.

10.1128/mBio.00989-18.7 TABLE S2  Metabolomics analysis of M. tuberculosis expressing Rv0077c showed several changes in cell envelope components. Metabolites in the shown M. tuberculosis strains were quantified by Metabolon, Inc. See Materials and Methods for sample preparation. Download TABLE S2, XLSX file, 0.3 MB. Copyright © 2018 Samanovic et al. 2018 Samanovic et al. This content is distributed under the terms of the Creative Commons Attribution 4.0 International license.

Acid-fast staining is primarily thought to be associated with mycolic acids; alterations in mycolic acid synthesis result in negative effects on cell growth *in vitro* and *in vivo* (18, 19). We used thin-layer and liquid or gas chromatography coupled to mass spectrometry to analyze the fatty acid, mycolic acid, and lipid contents of the WT and Rv0077c and Rv0078 mutant strains and observed no significant qualitative or quantitative differences between strains (see Fig. S4 and S5 and Table S3 in the supplemental material). In particular, the expression of Rv0077c did not conspicuously modify the chain length of mycolic acids, their cyclopropanation, or the relative abundance of keto- to α-mycolates and did not alter the wax ester and triglyceride contents of the strains that have previously been linked to acid-fast staining (19, 20).

10.1128/mBio.00989-18.4 FIG S4  Thin-layer chromatography (TLC) analysis of lipids and mycolic acids. (a) Lipid analyses. Equal amounts of lipid samples were loaded per TLC lane. TLCs were developed in the solvent systems chloroform-methanol-water at 20:4:0.5 by volume (solvent A), chloroform-methanol-water at 65:25:4 by volume (solvent B), or petroleum ether-ethyl acetate at 98:2 by volume, with three developments (solvent C), and revealed by spraying with cupric sulfate and heating. TMM, trehalose monomycolates; TDM, trehalose dimycolates; PE, phosphatidylethanolamine; CL, cardiolipin; PI, phosphatidylinositol; PIM_2_, phosphatidylinositol dimannosides; PIM_6_, phosphatidylinositol hexamannosides; SL, sulfolipids; TAG, triglycerides. (b) Mycolic acid analyses. Shown are the mycolic acids present in extractable lipids and those esterified to arabinogalactan (i.e., the cell-wall-bound mycolates). (a) “Alpha,” “methoxy,” and “keto” refer to the three types of mycolic acids produced by M. tuberculosis. The same amount of samples was loaded per lane. The thin-layer chromatograms were developed three times in the solvent system *n*-hexanes–ethyl acetate at 95:5 by volume and revealed by spraying with cupric sulfate and heating. MAMEs, mycolic acid methyl esters; FAMEs, fatty acid methyl esters. See Materials and Methods for details. Download FIG S4, PDF file, 1.6 MB. Copyright © 2018 Samanovic et al. 2018 Samanovic et al. This content is distributed under the terms of the Creative Commons Attribution 4.0 International license.

10.1128/mBio.00989-18.5 FIG S5  Mix scan of the mycolic acid region (18.6 to 25 min) of the LC-MS runs for the wild-type M. tuberculosis strain either treated with the DMSO carrier alone or treated with the iP compound, the Rv0077c transposon mutant, and the Rv0078 allelic exchange mutant. The average mass spectrum across the elution times of all three classes of mycolates (α, methoxy [M-], and keto [K-]) is presented for all four samples. The number of carbon atoms in each mycolic acid species is indicated above the corresponding peak. All four samples display comparable mycolic acid contents. Download FIG S5, PDF file, 0.5 MB. Copyright © 2018 Samanovic et al. 2018 Samanovic et al. This content is distributed under the terms of the Creative Commons Attribution 4.0 International license.

10.1128/mBio.00989-18.8 TABLE S3  GC-MS analysis of fatty acids of Rv0077c-expressing bacteria showed no conspicuous changes in several fatty acids. Fatty acid methyl esters were prepared from total extractable lipids as described in Materials and Methods and analyzed by GC-MS. The percentages of areas of the fatty acids to the total areas of the GC-MS chromatograms in each species are shown. Download TABLE S3, DOCX file, 0.1 MB. Copyright © 2018 Samanovic et al. 2018 Samanovic et al. This content is distributed under the terms of the Creative Commons Attribution 4.0 International license.

## DISCUSSION

Our studies are the first to demonstrate CKs induce robust and specific transcriptional and physiological changes in a bacterial species. M. tuberculosis treated with CK expressed high levels of Rv0077c, which altered its metabolome and staining properties. Despite these changes, we did not observe an effect on the susceptibility of these bacteria to antibiotics of different classes or on survival in mice. In addition we found the transcriptional regulator Rv0078 represses Rv0077c expression in the absence of CK, and we defined the operator to which Rv0078 binds. We determined that two dimers of Rv0078 bind to inverted repeats in the intergenic region between Rv0077c and Rv0078, which likely represses the expression of both genes.

In plants, CKs are sensed by membrane receptors related to the sensors of bacterial two-component systems (TCSs) (21–23). In these systems, CKs interact with membrane receptors via a CHASE (cyclases/histidine kinases associated sensing extracellular) domain (24, 25). As in bacteria, the ligand-receptor interaction stimulates a phosphorelay that ultimately leads to the phosphorylation of a response regulator protein, which either represses or activates gene expression. M. tuberculosis has at least 12 known TCSs, and none has a predicted CHASE domain (L. Aravind, February 2012, and I. Jouline, August 2017, personal communications). Thus, it remains to be determined if a TCS sensor protein is involved in CK signal transduction in M. tuberculosis.

It also remains to be determined what the natural ligand is for Rv0078; while SMARt-420 binds robustly to Rv0078 (14, 15), the cytokinin iP did not bind to this TetR-like regulator in our studies. This result may not be surprising when considering SMARt-420 and cytokinins bear no resemblance to each other. Furthermore, we could not cocrystallize iP with Rv0078 (data not shown). We hypothesize that the interaction of CK with an unknown receptor or enzyme leads to the synthesis of a small molecule (e.g., a lipid) that binds to Rv0078, resulting in the derepression of Rv0077c. This possibility may be supported by our observation that there is an increase in several phospholipids in iP-treated M. tuberculosis. We are currently working to identify factors required for CK-mediated gene induction.

Rv0077c is predicted to have an α/β-hydrolase fold (26), which can have a variety of substrate types (27). Based on our metabolomic and microscopy studies, we predict Rv0077c targets one or more components of the cell envelope. The identification of the natural target of Rv0077c may help provide new insight into the elusive molecular basis of acid-fastness of this human-exclusive pathogen. While it is well known that perturbations in mycolic acid synthesis result in loss of acid-fast staining (18, 19), our results may indicate that acid-fast staining can be affected by other changes in cell envelope chemistry. Alternatively, Rv0077c may have a subtle chemical effect that is undetectable using the available techniques for quantifying mycolic acids. Importantly, although our results show dramatic changes in acid-fast staining *in vitro*, we do not know if cytokinin signaling results in these alterations *in vivo*. It is possible that the production of Rv0077c only affects a partial pool of substrates within a subset of bacteria, the effects of which might not result in a total loss of staining as we observed *in vitro*.

While we did not observe any differences in bacterial burdens in mice infected with strains that either were disrupted for or constitutively expressed Rv0077c, it is possible that Rv0077c function might only be needed during very late or specific stages of infection. This idea may be supported by a previous observation that the cytokinin synthase *log* gene in Mycobacterium marinum is specifically expressed in late granulomas of infected frogs (28). Furthermore, mice might not provide an optimal model to observe a role for this pathway in tuberculosis.

A previous report suggested the expression of Rv0077c increases the sensitivity of M. tuberculosis to the antibiotic ETH. ETH must be activated by a monooxygenase, EthA, in order for it to be toxic to M. tuberculosis (16, 17). We found the expression of Rv0077c did not confer increased susceptibility to ETH or any other antibiotic we tested, an observation consistent with the likelihood that Rv0077c is not a monooxygenase. It is possible that the effects of SMARt-420 are growth condition dependent, that this molecule affects another pathway to increase the susceptibility of M. tuberculosis to ETH, or that another SMARt-420-induced enzyme and Rv0077c synergize to activate ETH (A. Baulard, personal communication). Irrespective of these possibilities, it is unlikely that Rv0077c has a considerable role in the activation of ETH. While a previous report named Rv0077c and Rv0078 “EthA2” and “EthR2,” respectively, we propose to rename them “LoaA” and “LoaR” for “loss of acid-fast staining A and repressor,” respectively, due to the lack of association of these proteins with ETH susceptibility.

Finally, our studies have opened the door to the possibility that numerous commensal and pathogenic microbes (including fungi) use cytokinins for intra- or interspecies communication in complex systems such as the gut microbiome. The identification of one or more CK receptors and the signal transduction pathway that leads to the induction of Rv0077c expression will likely lay the foundation for understanding CK signaling in hundreds of bacterial species.

## MATERIALS AND METHODS

### Bacterial strains, plasmids, primers, chemicals, and culture conditions.

The bacterial strains, plasmids, and primer sequences used in this study are listed in Table S1. All primers for cloning and sequencing were from Invitrogen, Inc. M. tuberculosis strains were grown in Middlebrook 7H9 broth (Difco) supplemented with 0.2% glycerol, 0.05% Tween 80, 0.5% fraction V of bovine serum albumin (BSA), 0.2% dextrose, and 0.085% sodium chloride (“7H9c”). M. tuberculosis cultures were grown without shaking in 25- or 75-cm^2^ vented flasks (Corning) at 37°C. 7H11 agar (Difco) supplemented with 0.5% glycerol and BBL TM Middlebrook OADC enrichment (BD) was used for growth on solid medium (“7H11”). M. tuberculosis was transformed as described previously (29). E. coli strains used for cloning and expression were grown in LB-Miller broth (Difco) at 37°C with aeration on a shaker or on LB agar. E. coli strains were chemically transformed as previously described (30). The final concentrations of antibiotics used for M. tuberculosis growth were as follows: kanamycin, 50 µg/ml; hygromycin, 50 µg/ml; and streptomycin, 25 µg/ml. The final concentrations for E. coli growth were as follows: hygromycin, 150 µg/ml; kanamycin, 100 µg/ml; and streptomycin, 50 µg/ml. AMP, adenine, and iP were purchased from Sigma. iPR, 2MeSiP, and 2MeSiPR were synthesized as previously described (31). The purity of the synthesized cytokinin and derivatives was >98% for each, as determined by high-performance liquid chromatography and mass spectrometry.

The Rv0077c::MycoMarT7 mutant was isolated from a library of ordered transposon insertion mutants as previously described (3, 32). The ΔRv0078::*hyg* mutant was made by deletion-disruption mutagenesis as described in detail elsewhere using pYUB854 (33, 34). All mutants were confirmed by PCR using primers that annealed to sequence beyond the region used for making allelic exchange plasmids.

### Protein purification and immunoblotting.

DNA sequence encompassing the full-length Rv0078 or Rv0077c gene was cloned into pET24b(+) vector using primers listed in Table S1. Recombinant proteins were produced in E. coli ER2566 and purified under native conditions for Rv0078 and denaturing conditions for Rv0077c according to the manufacturer’s specifications (Qiagen). Polyclonal rabbit antibodies were raised by Covance (Denver, PA). For all immunoblots, cell lysates or purified proteins were separated by sodium dodecyl sulfate-polyacrylamide gel electrophoresis (SDS-PAGE), transferred to nitrocellulose, and incubated with rabbit polyclonal antibodies to the protein of interest at a 1:1,000 dilution in 3% bovine serum albumin in Tris-buffered saline–Tween (TBST: 25 mM Tris-HCl [pH 7.4], 125 mM NaCl, 0.05% Tween 20 [pH 7.4]). Equal loading was determined by stripping the nitrocellulose membranes with 0.2 N NaOH for 5 min, rinsing, blocking, and incubating the nitrocellulose with polyclonal rabbit antibodies to dihydrolipoamide acyltransferase (DlaT) (35). Horseradish peroxidase-conjugated anti-rabbit antibody (GE-Amersham Biosciences, Inc.) was used for chemiluminescent detection (SuperSignal West Pico; Thermo Fisher Scientific).

For crystallography studies of Rv0078-His_6_, bacteria were grown at 37°C to an optical density at 600 nm (OD_600_) of 0.5 to 0.6 before being induced with 0.5 mM IPTG (isopropyl-β-d-thiogalactopyranoside) and incubated at 16°C overnight. After being harvesting by centrifugation, cells were lysed by passing through a microfluidizer cell disrupter in a mixture of 10 mM potassium phosphate (pH 8.0), 10 mM imidazole, and 500 mM NaCl. The homogenate was clarified by spinning at 27,000 × *g*, and the supernatant was applied to a HiTrap-Ni column (GE Healthcare) preequilibrated with the lysis buffer. Histidine-tagged protein was eluted with a 10 to 300 mM imidazole gradient in 10 mM potassium phosphate (pH 8.0) containing 300 mM NaCl. The Rv0078 fractions were further purified by a Superdex 75 column (16 by 1,000 mm; GE Healthcare) preequilibrated with 20 mM potassium phosphate (pH 8.0) and 300 mM NaCl. The purified Rv0078 was concentrated to 40 mg/ml for the crystallization screen.

### RNA-Seq and 5′ RACE.

Three biological replicate cultures of WT M. tuberculosis were grown to an OD_580_ of ~1 and incubated in 100 µM iP or DMSO (control) for 5 h. Cells were harvested and RNA was purified as described previously (33). Briefly, an equal volume of 4 M guanidinium isothiocyanate, 0.5% sodium *N*-lauryl sarcosine, and 25 mM trisodium citrate solution was added to cultures to arrest transcription. RNA was isolated with Trizol reagent (Invitrogen) and further purified using RNeasy Miniprep kits and DNase I (Qiagen). Transcriptome profiling by RNA-Seq was performed, and the results were analyzed as follows. RNA from M. tuberculosis cultures was extracted for library construction. Libraries were constructed and bar-coded with the Epicentre ScriptSeq Complete Gold Low Input (Illumina, Inc.) and sequenced on an Illumina HiSeq 2000 sequencer using version 3 reagents. Unique sequence reads were mapped to the corresponding reference genome, and reads per kilobase per million (RPKM) values were calculated in CLC (version 7.0.4). Genes with significantly different RPKM values were identified using the Significant Analysis for Microarray (SAM) statistical analysis component of MeV (36).

5′ RACE was performed as described by the manufacturer (Invitrogen). Briefly, 1 µg of RNA was used as the template for cDNA production using a reverse primer 150 to 300 bp downstream of annotated translational start sites. A 3′ poly(C) tail was added to cDNA by recombinant Tdt. The cDNA was then amplified using a nested reverse primer and a primer that anneals to the poly(C) tail. Products were cloned and sequenced. Likely transcriptional start sites were selected based on the clones that had the most nucleotide sequence upstream of the start codon.

### EMSA.

A series of double-stranded DNA probes consisting of sequences in the intergenic region between Rv0077c and Rv0078 were generated by annealing two complementary oligonucleotides and 5′-end IRDye700-labeled 14-nucleotide oligomers (5′-dye-GTGCCCTGGTCTGG-3′) (Integrated DNA Technologies, Inc.). Binding assays were performed by incubating 100 nM probes and various concentrations of Rv0078 at room temperature for 30 min in a mixture of 20 mM HEPES (pH 7.5), 3 mM dithiothreitol (DTT), 0.1 mM EDTA, 100 mM KCl, 5% glycerol, 5 mg/ml bovine serum albumin (BSA), 10 mM MgCl_2_, and 0.25% Tween 20 and were subsequently resolved in 6% polyacrylamide gels in 0.5× Tris-borate-EDTA (TBE) buffer. Mobility shifts of protein-DNA complex were visualized in Li-Cor Odyssey imager.

### Crystallization and structure determination.

DNA-free Rv0078 crystals were obtained by screening at 20°C using the sitting-drop vapor diffusion method. The C2 space group crystals were grown in 0.1 M sodium cacodylate, pH 6.4, and 1.3 M lithium-sulfate. SeMet substituted Rv0078 crystals with C2 space group were grown in a mixture of 0.1 M sodium cacodylate (pH 6.6), 1.3 M lithium sulfate, 0.2 M magnesium sulfate, and 2% polyethylene glycol 400 (PEG 400). Diffraction data to a resolution of 1.85 Å were collected at the Lilly Research Laboratories Collaborative Access Team (LRL-CAT) beamline of Advanced Photon Source (APS), Argonne National Laboratory, and were processed with Mosflm software (37). The program Hybrid-Substructure-Search in the Phenix package was used to locate the Se sites, and the initial phasing was carried out using the program Autosol of Phenix. The 2.7-Å map phased by the SAD method allowed us to build the Rv0078 model unambiguously. The native Rv0078 structure was subsequently determined by the program PHASER using SeMet-substituted Rv0078 as the initial search model. To obtain the Rv0078-DNA crystals, purified Rv0078 was cocrystallized with a 23-mer DNA duplex (5′-TTTACAAGCAGACTGCCGGTAAC-3′) at a molar ratio of 2:1 (protein dimer to DNA) in the presence of 150 mM MgCl_2_. The DNA-bound Rv0078 crystals were grown in the buffer containing only 0.2 M magnesium formate. Diffraction data to 3.0 Å were collected at the Life Sciences Collaborative Access Team (LS-CAT) beamline of APS and were processed with Mosflm. The Rv0078 DNA structure was determined by PHASER using DNA-free structure as the search model. All the refinements were performed using Phenix-refine (38). The statistics are provided in Table 3.

### Mass spectrometry of fatty acids copurified with Rv0078.

Fatty acids were extracted from 5 mg Rv0078 that was purified from E. coli and analyzed as described previously (39). Separation and identification of the fatty acid (FA) methyl esters were performed on an HP5975 gas chromatograph-mass spectrometer (Hewlett-Packard) fitted with a 60-m by 250-µm SP-2340 capillary column (Supelco) with helium as the carrier gas.

### ITC.

The isothermal calorimetry (ITC) experiment was performed in a Microcal PEAQ-ITC at 25°C. The stirring speed was 750 rpm, and the interval between each titration was 150 s. The concentration of Rv0078 in the reaction cell was 25 µM, and the concentration of the titration DNA ligand was 500 µM. The recorded thermal data were analyzed using Microcal PEAQ-ITC analysis software.

### IC_50_ determination.

To determine the IC_50_ of each antibiotic, M. tuberculosis strains were grown to an OD_580_ of ~0.7 and diluted into fresh medium to an OD_580_ of 0.02. Diluted cultures were transferred to a 96-well microtiter plate containing triplicate two-fold serial dilutions of antibiotic. Cell-wall-active antibiotics (vancomycin and meropenem) were supplemented at all concentrations with 5 µg/ml potassium clavulanate to inhibit the intrinsic β-lactamase activity of M. tuberculosis. After 5 days of incubation at 37°C, growth in each well was measured by OD_580_. IC_50_ values were interpolated from a nonlinear least-squares fit of log_2_-transformed OD_580_ measurements. Data are representative of two independent experiments. Antibiotics were purchased from Sigma-Aldrich (clavulanate, ethionamide, meropenem, rifampin, and vancomycin) or Thermo-Fisher Scientific (ciprofloxacin, ethambutol, isoniazid, norfloxacin, and streptomycin).

### Mouse infections.

Mouse infections were performed essentially as described previously (3). Seven- to 9-week-old female C57BL6/J mice (The Jackson Laboratory) were infected by aerosol to deliver ~200 bacilli per mouse, using a Glas-Col inhalation exposure system (Terre Haute, IN). The strains used were the WT strain (MHD761 or MHD794), an Rv0077c mutant (MHD1086), an Rv0077c mutant complemented strain (MHD1077), an Rv0078 mutant (MHD1315), an Rv0078 mutant strain complemented with WT Rv0078 (MHD1318), and an Rv0078_W100R_ mutant (MHD1316). This study was performed in strict accordance with the recommendations in the *Guide for the Care and Use of Laboratory Animals* of the National Institutes for Health. Mice were humanely euthanized according to an approved Institutional Animal Care and Use Committee protocol at New York University School of Medicine. Lungs and spleens were harvested and homogenized in phosphate-buffered saline (PBS)–0.05% Tween 80 at the indicated time points to determine bacterial CFU.

### Metabolomic analysis of M. tuberculosis cell lysates.

Four independent cultures of each analyzed strain were grown in 7H9 to an OD_580_ of ~0.7 and treated with iP in DMSO at a final concentration of 100 µM or an equal volume of DMSO for 24 h. Bacteria were harvested the next day at an OD_580_ of ~1. Sixty-five OD equivalents per replicate were processed by chloroform-methanol extraction (8, 40). Metabolomic profiling was performed by Metabolon, Inc.

### Staining and microscopy.

M. tuberculosis strains were grown to mid-logarithmic phase (OD_580_ of ~0.5 to 0.7). Five microliters of culture was spotted onto glass slides and heat fixed over a flame or on a heat block (2 min, 95°C). Staining was performed according to the method of Ziehl-Neelsen as per the manufacturer’s instructions (BD Stain Kit ZN). Images were acquired on a Zeiss Axio Observer with a Plan-Apochromat 63×/1.4 oil lens. Images were taken with an Axiocam503 camera at the New York University Langone Medical Center (NYULMC) Microscopy Laboratory.

### Analysis of total lipids, mycolic acids, and shorter-chain fatty acids.

For lipid analysis, 400-ml cultures were grown up to an OD_580_ of ~0.7 and treated with iP in DMSO at a final concentration of 100 µM or an equal volume of DMSO only for 24 h. Four hundred milliliters of Rv0077c and Rv0078 cultures was treated with DMSO only. Cells were washed three times in Dulbecco’s phosphate-buffered saline (DPBS) and heated at 100°C for 45 min for sterilization before freezing at −20°C. Extraction of total lipids from bacterial cells and preparation of fatty acid and mycolic acid methyl esters from extractable lipids and delipidated cells followed earlier procedures (41). Total lipids and fatty acid/mycolic acid methyl esters were analyzed by one- and two-dimensional thin-layer chromatography (TLC) in a variety of solvent systems on aluminum-backed silica gel 60-precoated plates F_254_ (E. Merck). TLC plates were revealed by spraying with cupric sulfate (10% in an 8% phosphoric acid solution) and heating. Alternatively, total lipids were run in both positive and negative mode, with the released fatty acids/mycolic acids run in negative mode only, on a high-resolution Agilent 6220 time of flight (TOF) mass spectrometer interfaced to a liquid chromatograph as described previously (42, 43). Data files were analyzed with Agilent’s Mass Hunter workstation software, and most compounds were identified using a database of *M. tuberculosis* lipids developed in house (42). Fatty acid methyl esters from extractable lipids were treated with 3 M HCl in CH_3_OH (Supelco) overnight at 80°C, dried, and dissolved in *n*-hexane(s) prior to GC-MS analysis. GC-MS analyses of fatty acid methyl esters were carried out using a Trace 1310 gas chromatograph (Thermo Fisher) equipped with a TSQ 8000 Evo triple quadrupole in the electron impact mode and scanning from *m*/*z* 70 to *m*/*z* 1,000 over 0.8 s. Helium was used as the carrier gas with a flow rate of 1 ml per min. The samples were run on a ZB-5HT column (15 m by 0.25-mm inside diameter [i.d.]) (Zebron). The injector (splitless mode) was set for 300°C (350°C for mycolic acid methyl esters). The oven temperature was held at 60°C for 2 min, programmed at 20°C per min to 375°C, followed by a 10-min hold. The data analyses were carried out on a Chromeleon data station.

## References

[1] WHO 2017 Tuberculosis. World Health Organization, Geneva, Switzerland http://www.who.int/mediacentre/factsheets/fs104/en/.

[2] Cerda-Maira FA, Pearce MJ, Fuortes M, Bishai WR, Hubbard SR, Darwin KH 2010 Molecular analysis of the prokaryotic ubiquitin-like protein (Pup) conjugation pathway in Mycobacterium tuberculosis. Mol Microbiol 77:1123–1135. doi:10.1111/j.1365-2958.2010.07276.x. 20636328 PMC2975802

[3] Darwin KH, Ehrt S, Gutierrez-Ramos JC, Weich N, Nathan CF 2003 The proteasome of Mycobacterium tuberculosis is required for resistance to nitric oxide. Science 302:1963–1966. doi:10.1126/science.1091176. 14671303

[4] Gandotra S, Lebron MB, Ehrt S 2010 The Mycobacterium tuberculosis proteasome active site threonine is essential for persistence yet dispensable for replication and resistance to nitric oxide. PLoS Pathog 6:e1001040. doi:10.1371/journal.ppat.1001040. 20711362 PMC2920845

[5] Gandotra S, Schnappinger D, Monteleone M, Hillen W, Ehrt S 2007 In vivo gene silencing identifies the Mycobacterium tuberculosis proteasome as essential for the bacteria to persist in mice. Nat Med 13:1515–1520. doi:10.1038/nm1683. 18059281 PMC3174471

[6] Lamichhane G, Raghunand TR, Morrison NE, Woolwine SC, Tyagi S, Kandavelou K, Bishai WR 2006 Deletion of a Mycobacterium tuberculosis proteasomal ATPase homologue gene produces a slow-growing strain that persists in host tissues. J Infect Dis 194:1233–1240. doi:10.1086/508288. 17041849

[7] Lin G, Li D, de Carvalho LP, Deng H, Tao H, Vogt G, Wu K, Schneider J, Chidawanyika T, Warren JD, Li H, Nathan C 2009 Inhibitors selective for mycobacterial versus human proteasomes. Nature 461:621–626. doi:10.1038/nature08357. 19759536 PMC3172082

[8] Samanovic MI, Tu S, Novák O, Iyer LM, McAllister FE, Aravind L, Gygi SP, Hubbard SR, Strnad M, Darwin KH 2015 Proteasomal control of cytokinin synthesis protects Mycobacterium tuberculosis against nitric oxide. Mol Cell 57:984–994. doi:10.1016/j.molcel.2015.01.024. 25728768 PMC4369403

[9] Sakakibara H 2006 Cytokinins: activity, biosynthesis, and translocation. Annu Rev Plant Biol 57:431–449. doi:10.1146/annurev.arplant.57.032905.105231. 16669769

[10] Frébort I, Kowalska M, Hluska T, Frébortová J, Galuszka P 2011 Evolution of cytokinin biosynthesis and degradation. J Exp Bot 62:2431–2452. doi:10.1093/jxb/err004. 21321050

[11] Lechat P, Hummel L, Rousseau S, Moszer I 2008 GenoList: an integrated environment for comparative analysis of microbial genomes. Nucleic Acids Res 36:D469–D474. doi:10.1093/nar/gkm1042. 18032431 PMC2238853

[12] Brosch R, Gordon SV, Marmiesse M, Brodin P, Buchrieser C, Eiglmeier K, Garnier T, Gutierrez C, Hewinson G, Kremer K, Parsons LM, Pym AS, Samper S, van Soolingen D, Cole ST 2002 A new evolutionary scenario for the Mycobacterium tuberculosis complex. Proc Natl Acad Sci U S A 99:3684–3689. doi:10.1073/pnas.052548299. 11891304 PMC122584

[13] Orth P, Schnappinger D, Hillen W, Saenger W, Hinrichs W 2000 Structural basis of gene regulation by the tetracycline inducible Tet repressor-operator system. Nat Struct Biol 7:215–219. doi:10.1038/73324. 10700280

[14] Wohlkönig A, Remaut H, Moune M, Tanina A, Meyer F, Desroses M, Steyaert J, Willand N, Baulard AR, Wintjens R 2017 Structural analysis of the interaction between spiroisoxazoline SMARt-420 and the Mycobacterium tuberculosis repressor EthR2. Biochem Biophys Res Commun 487:403–408. doi:10.1016/j.bbrc.2017.04.074. 28416386

[15] Blondiaux N, Moune M, Desroses M, Frita R, Flipo M, Mathys V, Soetaert K, Kiass M, Delorme V, Djaout K, Trebosc V, Kemmer C, Wintjens R, Wohlkönig A, Antoine R, Huot L, Hot D, Coscolla M, Feldmann J, Gagneux S, Locht C, Brodin P, Gitzinger M, Déprez B, Willand N, Baulard AR 2017 Reversion of antibiotic resistance in Mycobacterium tuberculosis by spiroisoxazoline SMARt-420. Science 355:1206–1211. doi:10.1126/science.aag1006. 28302858

[16] DeBarber AE, Mdluli K, Bosman M, Bekker LG, Barry CE III 2000 Ethionamide activation and sensitivity in multidrug-resistant Mycobacterium tuberculosis. Proc Natl Acad Sci U S A 97:9677–9682. doi:10.1073/pnas.97.17.9677. 10944230 PMC16924

[17] Vannelli TA, Dykman A, Ortiz de Montellano PR 2002 The antituberculosis drug ethionamide is activated by a flavoprotein monooxygenase. J Biol Chem 277:12824–12829. doi:10.1074/jbc.M110751200. 11823459

[18] Barkan D, Liu Z, Sacchettini JC, Glickman MS 2009 Mycolic acid cyclopropanation is essential for viability, drug resistance, and cell wall integrity of Mycobacterium tuberculosis. Chem Biol 16:499–509. doi:10.1016/j.chembiol.2009.04.001. 19477414 PMC2731493

[19] Bhatt A, Fujiwara N, Bhatt K, Gurcha SS, Kremer L, Chen B, Chan J, Porcelli SA, Kobayashi K, Besra GS, Jacobs WR Jr. 2007 Deletion of kasB in Mycobacterium tuberculosis causes loss of acid-fastness and subclinical latent tuberculosis in immunocompetent mice. Proc Natl Acad Sci U S A 104:5157–5162. doi:10.1073/pnas.0608654104. 17360388 PMC1829279

[20] Deb C, Lee CM, Dubey VS, Daniel J, Abomoelak B, Sirakova TD, Pawar S, Rogers L, Kolattukudy PE 2009 A novel in vitro multiple-stress dormancy model for Mycobacterium tuberculosis generates a lipid-loaded, drug-tolerant, dormant pathogen. PLoS One 4:e6077. doi:10.1371/journal.pone.0006077. 19562030 PMC2698117

[21] To JP, Kieber JJ 2008 Cytokinin signaling: two-components and more. Trends Plant Sci 13:85–92. doi:10.1016/j.tplants.2007.11.005. 18262459

[22] Inoue T, Higuchi M, Hashimoto Y, Seki M, Kobayashi M, Kato T, Tabata S, Shinozaki K, Kakimoto T 2001 Identification of CRE1 as a cytokinin receptor from Arabidopsis. Nature 409:1060–1063. doi:10.1038/35059117. 11234017

[23] Steklov MY, Lomin SN, Osolodkin DI, Romanov GA 2013 Structural basis for cytokinin receptor signaling: an evolutionary approach. Plant Cell Rep 32:781–793. doi:10.1007/s00299-013-1408-3. 23525743

[24] Mougel C, Zhulin IB 2001 CHASE: an extracellular sensing domain common to transmembrane receptors from prokaryotes, lower eukaryotes and plants. Trends Biochem Sci 26:582–584. doi:10.1016/S0968-0004(01)01969-7. 11590001

[25] Anantharaman V, Aravind L 2001 The CHASE domain: a predicted ligand-binding module in plant cytokinin receptors and other eukaryotic and bacterial receptors. Trends Biochem Sci 26:579–582. doi:10.1016/S0968-0004(01)01968-5. 11590000

[26] Söding J, Biegert A, Lupas AN 2005 The HHpred interactive server for protein homology detection and structure prediction. Nucleic Acids Res 33:W244–W248. doi:10.1093/nar/gki408. 15980461 PMC1160169

[27] Nardini M, Dijkstra BW 1999 Alpha/beta hydrolase fold enzymes: the family keeps growing. Curr Opin Struct Biol 9:732–737. doi:10.1016/S0959-440X(99)00037-8. 10607665

[28] Ramakrishnan L, Federspiel NA, Falkow S 2000 Granuloma-specific expression of Mycobacterium virulence proteins from the glycine-rich PE-PGRS family. Science 288:1436–1439. doi:10.1126/science.288.5470.1436. 10827956

[29] Hatfull GF, Jacobs WRJ 2000 Molecular genetics of mycobacteria. ASM Press, Washington, DC.

[30] Sambrook J, Maniatis T, Fritsch E 1989 Molecular cloning: a laboratory manual, 2nd ed. Cold Spring Harbor Laboratory Press, Cold Spring Harbor, NY.

[31] Sugiyama T, Hashizume T 1978 Ribosylation of 6-chloropurine and its 2-methylthio derivative by a fusion procedure using iodin. Agric Biol Chem 42:1791–1792.

[32] Festa RA, Pearce MJ, Darwin KH 2007 Characterization of the proteasome accessory factor (paf) operon in Mycobacterium tuberculosis. J Bacteriol 189:3044–3050. doi:10.1128/JB.01597-06. 17277063 PMC1855869

[33] Festa RA, Jones MB, Butler-Wu S, Sinsimer D, Gerads R, Bishai WR, Peterson SN, Darwin KH 2011 A novel copper-responsive regulon in Mycobacterium tuberculosis. Mol Microbiol 79:133–148. doi:10.1111/j.1365-2958.2010.07431.x. 21166899 PMC3052634

[34] Bardarov S, Bardarov S Jr, Pavelka MS Jr, Sambandamurthy V, Larsen M, Tufariello J, Chan J, Hatfull G, Jacobs WR Jr. 2002 Specialized transduction: an efficient method for generating marked and unmarked targeted gene disruptions in Mycobacterium tuberculosis, M. bovis BCG and M. smegmatis. Microbiology 148:3007–3017. doi:10.1099/00221287-148-10-3007. 12368434

[35] Tian J, Bryk R, Shi S, Erdjument-Bromage H, Tempst P, Nathan C 2005 Mycobacterium tuberculosis appears to lack alpha-ketoglutarate dehydrogenase and encodes pyruvate dehydrogenase in widely separated genes. Mol Microbiol 57:859–868. doi:10.1111/j.1365-2958.2005.04741.x. 16045627

[36] Saeed AI, Sharov V, White J, Li J, Liang W, Bhagabati N, Braisted J, Klapa M, Currier T, Thiagarajan M, Sturn A, Snuffin M, Rezantsev A, Popov D, Ryltsov A, Kostukovich E, Borisovsky I, Liu Z, Vinsavich A, Trush V, Quackenbush J 2003 TM4: a free, open-source system for microarray data management and analysis. Biotechniques 34:374–378. 12613259 10.2144/03342mt01

[37] Winn MD, Ballard CC, Cowtan KD, Dodson EJ, Emsley P, Evans PR, Keegan RM, Krissinel EB, Leslie AG, McCoy A, McNicholas SJ, Murshudov GN, Pannu NS, Potterton EA, Powell HR, Read RJ, Vagin A, Wilson KS 2011 Overview of the CCP4 suite and current developments. Acta Crystallogr D Biol Crystallogr 67:235–242. doi:10.1107/S0907444910045749. 21460441 PMC3069738

[38] Adams PD, Afonine PV, Bunkóczi G, Chen VB, Davis IW, Echols N, Headd JJ, Hung LW, Kapral GJ, Grosse-Kunstleve RW, McCoy AJ, Moriarty NW, Oeffner R, Read RJ, Richardson DC, Richardson JS, Terwilliger TC, Zwart PH 2010 PHENIX: a comprehensive Python-based system for macromolecular structure solution. Acta Crystallogr D Biol Crystallogr 66:213–221. doi:10.1107/S0907444909052925. 20124702 PMC2815670

[39] Fan J, Yan C, Xu C 2013 Phospholipid:diacylglycerol acyltransferase-mediated triacylglycerol biosynthesis is crucial for protection against fatty acid-induced cell death in growing tissues of Arabidopsis. Plant J 76:930–942. doi:10.1111/tpj.12343. 24118513

[40] Layre E, Sweet L, Hong S, Madigan CA, Desjardins D, Young DC, Cheng TY, Annand JW, Kim K, Shamputa IC, McConnell MJ, Debono CA, Behar SM, Minnaard AJ, Murray M, Barry CE III, Matsunaga I, Moody DB 2011 A comparative lipidomics platform for chemotaxonomic analysis of Mycobacterium tuberculosis. Chem Biol 18:1537–1549. doi:10.1016/j.chembiol.2011.10.013. 22195556 PMC3407843

[41] Stadthagen G, Korduláková J, Griffin R, Constant P, Bottová I, Barilone N, Gicquel B, Daffé M, Jackson M 2005 p-Hydroxybenzoic acid synthesis in Mycobacterium tuberculosis. J Biol Chem 280:40699–40706. doi:10.1074/jbc.M508332200. 16210318

[42] Sartain MJ, Dick DL, Rithner CD, Crick DC, Belisle JT 2011 Lipidomic analyses of Mycobacterium tuberculosis based on accurate mass measurements and the novel “Mtb LipidDB”. J Lipid Res 52:861–872. doi:10.1194/jlr.M010363. 21285232 PMC3073466

[43] Bhamidi S, Shi L, Chatterjee D, Belisle JT, Crick DC, McNeil MR 2012 A bioanalytical method to determine the cell wall composition of Mycobacterium tuberculosis grown in vivo. Anal Biochem 421:240–249. doi:10.1016/j.ab.2011.10.046. 22107887

[44] Shi X, Festa RA, Ioerger TR, Butler-Wu S, Sacchettini JC, Darwin KH, Samanovic MI 2014 The copper-responsive RicR regulon contributes to Mycobacterium tuberculosis virulence. mBio 5:e00876-13. doi:10.1128/mBio.00876-13. 24549843 PMC3944814

[45] Chong YH, Jung JM, Choi W, Park CW, Choi KS, Suh YH 1994 Bacterial expression, purification of full length and carboxyl terminal fragment of Alzheimer amyloid precursor protein and their proteolytic processing by thrombin. Life Sci 54:1259–1268. doi:10.1016/0024-3205(94)00853-1. 8164508

[46] Stover CK, de la Cruz VF, Fuerst TR, Burlein JE, Benson LA, Bennett LT, Bansal GP, Young JF, Lee MH, Hatfull GF, Snapper SB, Barletta RG, Jacobs WR Jr, Bloom BR 1991 New use of BCG for recombinant vaccines. Nature 351:456–460. doi:10.1038/351456a0. 1904554

