# Supplementary material for: Cytokinin Signaling in Mycobacterium tuberculosis
Source: mBio. 2018 Jun 19;9(3):e00989-18. doi: 10.1128/mBio.00989-18 (PMC6016246; doi:10.1128/mBio.00989-18)
Supplement: FIG S1 [file mbo003183940sf1.pdf]

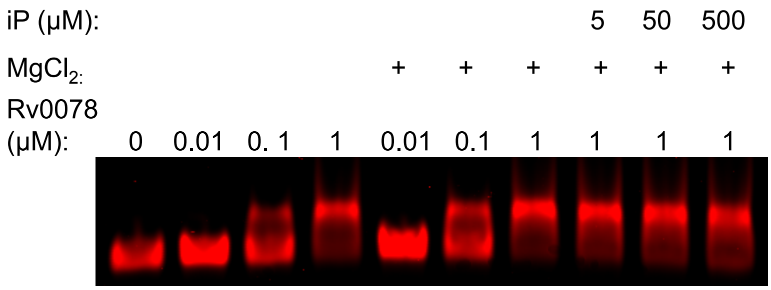

Fig. S1 . The cytokinin iP cannot dissociate Rv0078 from DNA. 6% TBE gel was imaged using a LI-COR Odyssey imager. 10 mM $MgCl_2$ improved DNA binding.
