# Supplementary material for: Cytokinin Signaling in Mycobacterium tuberculosis
Source: mBio. 2018 Jun 19;9(3):e00989-18. doi: 10.1128/mBio.00989-18 (PMC6016246; doi:10.1128/mBio.00989-18)
Supplement: FIG S2 [file mbo003183940sf2.pdf]

Samanovic, Hsu et al, Fig. S2

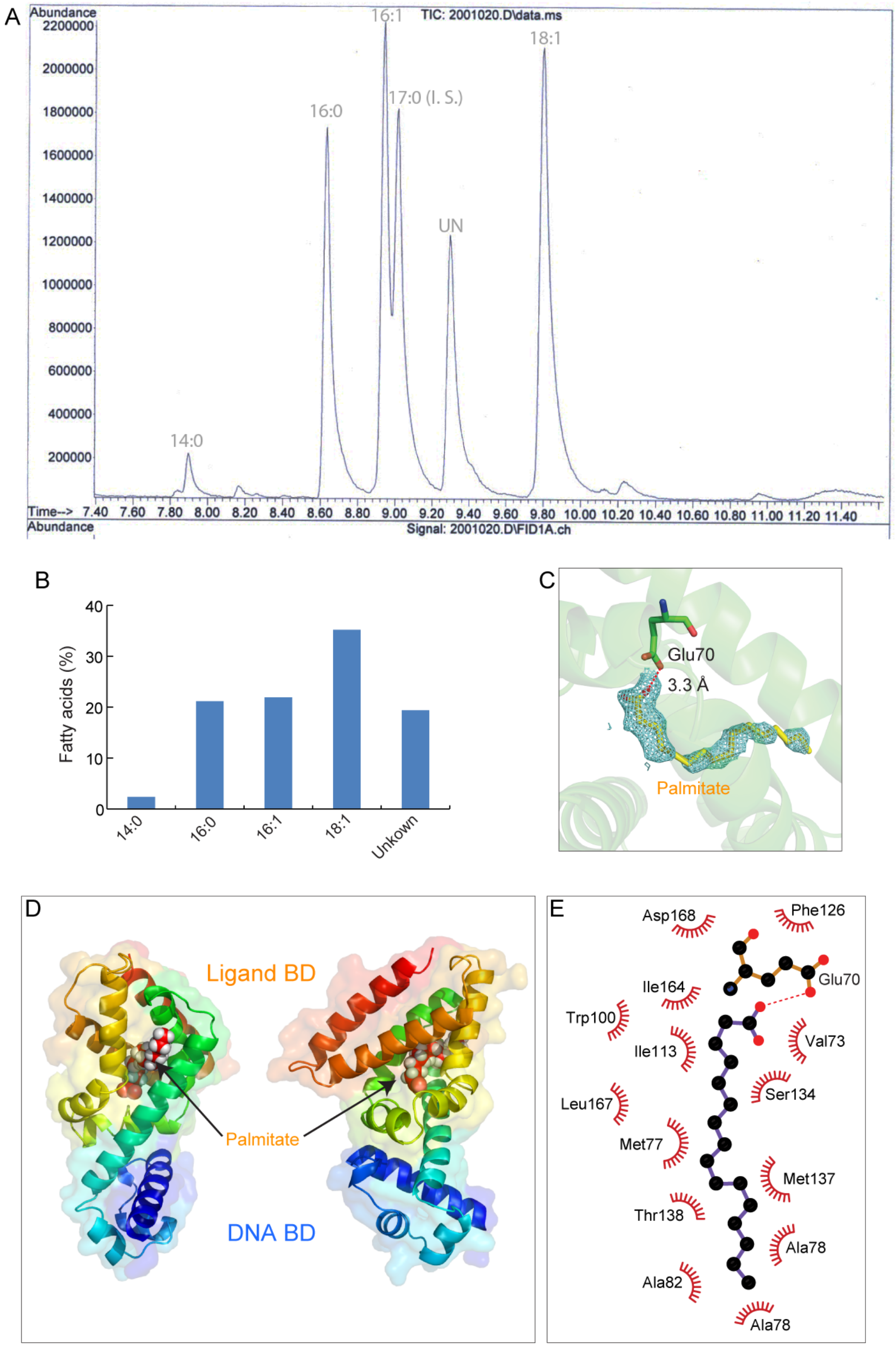

Fig. S2: Rv0078 purified from *E. coli* binds fatty acids. (**A**) GS-MS of fatty acids extracted from *E. coli*-expressed and purified Rv0078. Each identified fatty acid peak is labeled by the total number of carbon followed by the number of unsaturated C-C bond. I.S.: internal standard. UN: unknown peak. (**B**) A quantification of the relative amount of fatty acids bound to Rv0078. (**C**) A palmitate fits the 2Fo-Fc density map in the ligand binding pocket. The density is contoured at 1σ in blue mesh. (**D**) The structure of Rv0078 bound to a fatty acid. Protein is shown in rainbow cartoon, and the ligand in spheres. Only one monomer of the dimer is shown. (**E**) Except for an H bond with Glu-70, the palmitate is surrounded by hydrophobic residues of the ligand-binding pocket. This panel was generated with the program Ligplot+ (Laskowski R A, Swindells M B (2011) J. Chem. Inf. Model., 51, 2778-2786).
