# Supplementary material for: Cytokinin Signaling in Mycobacterium tuberculosis
Source: mBio. 2018 Jun 19;9(3):e00989-18. doi: 10.1128/mBio.00989-18 (PMC6016246; doi:10.1128/mBio.00989-18)
Supplement: FIG S3 [file mbo003183940sf3.pdf]

Samanovic, Hsu et al, Fig. S3

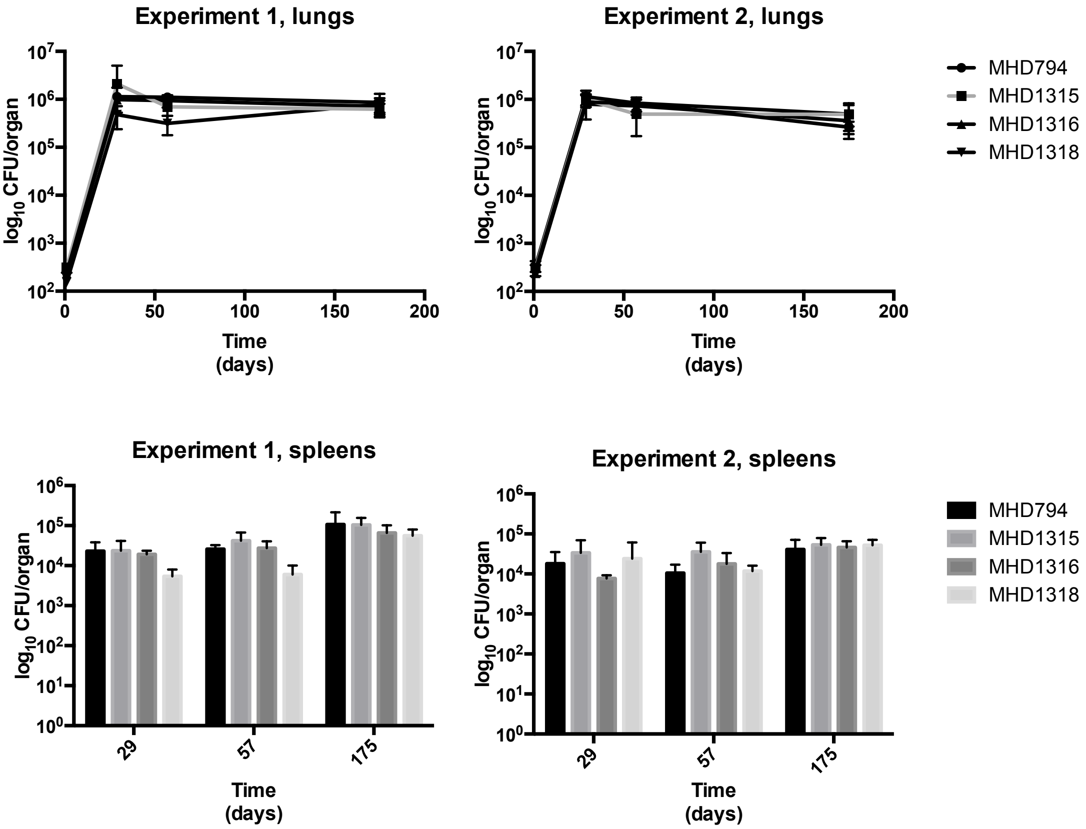

Fig. S3: An Rv0078 deletion-disruption mutant, which overexpresses Rv0077c, has no long term virulence defect in C57BL6/J mice. For each experiment, 16 mice were infected with 200-400 colony forming units (CFU) of each strain (64 mice/experiment) by the aerosol route of infection. See Methods for details. MHD794 = WT with empty vector; MHD1315 = ΔRv0078::*hyg* with empty vector; MHD1316 = ΔRv0078::*hyg* with Rv0078W100R; MHD1318 = ΔRv0078::*hyg* complemented with WT Rv0078. Error bars indicate the standard error of the mean.
