# Supplementary material for: Cytokinin Signaling in Mycobacterium tuberculosis
Source: mBio. 2018 Jun 19;9(3):e00989-18. doi: 10.1128/mBio.00989-18 (PMC6016246; doi:10.1128/mBio.00989-18)
Supplement: FIG S4 [file mbo003183940sf4.pdf]

Samanovic, Hsu et al, Fig. S4

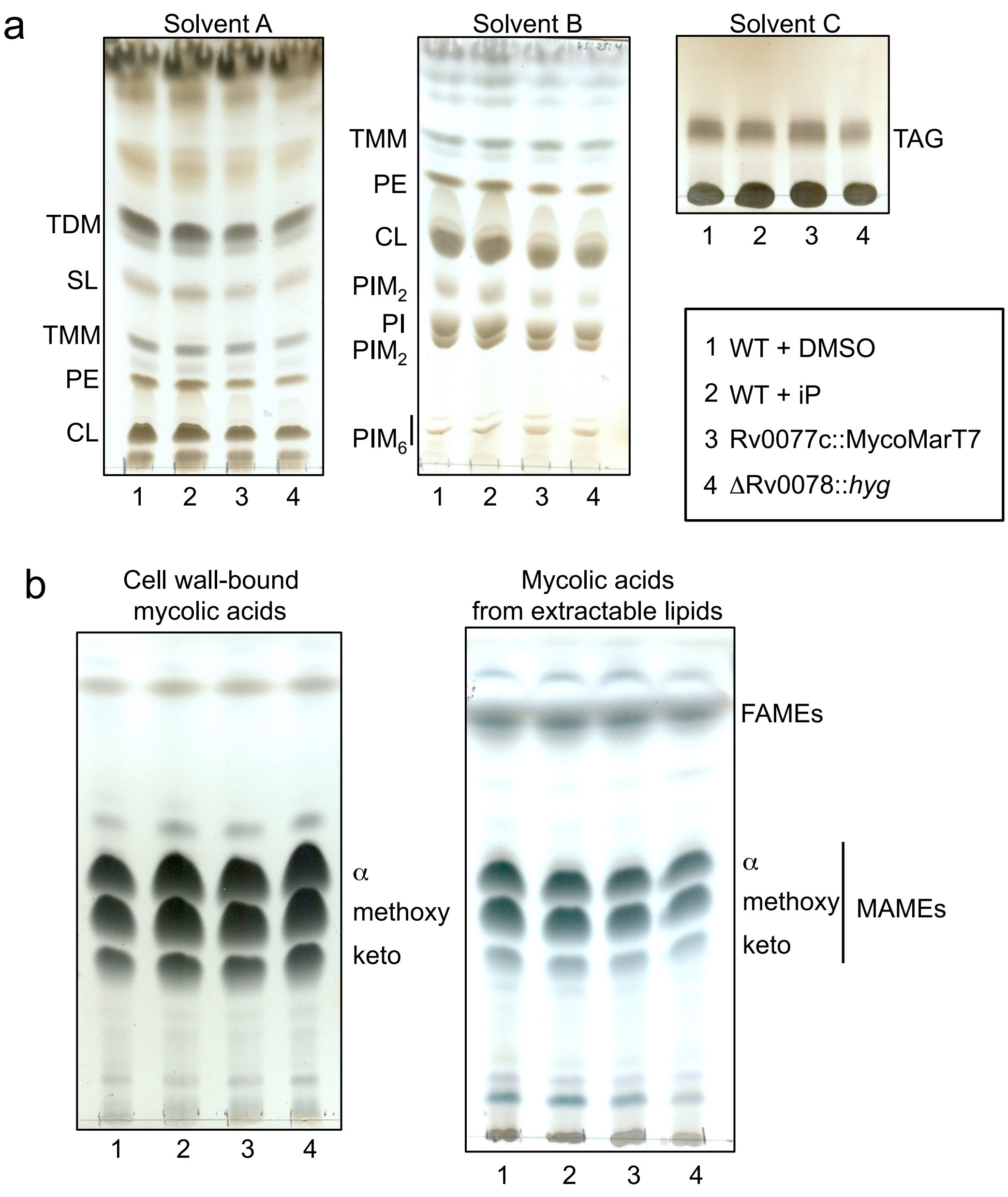

Fig. S4: Thin-layer chromatography (TLC) analysis of lipids and mycolic acids. a, Lipid analyses. Equal amounts of lipid samples were loaded per TLC lane. TLCs were developed in the solvent systems [chloroform:methanol:water] (20:4:0.5, by vol.) (solvent A), [chloroform:methanol:water] (65:25:4, by vol.) (solvent B) or petroleum ether: ethyl acetate (98:2, by vol.; three developments) (solvent C) and revealed by spraying with cupric sulfate and heating. TMM, trehalose monomycolates; TDM, trehalose dimycolates; PE, phosphatidylethanolamine; CL, cardiolipin; PI, phosphatidylinositol; PIM_2, phosphatidylinositol dimannosides; PIM_6, phosphatidylinositol hexamannosides; SL, sulfolipids; TAG, triglycerides. **b,** Mycolic acid analyses. The mycolic acids present in extractable lipids and those esterified to arabinogalactan (i.e., the cell wall-bound mycolates). α, methoxy- and keto- refer to the three types of mycolic acids produced by *M. tuberculosis*. The same amount of samples was loaded per lane. The TLCs were developed thrice in the solvent system [*n*-hexanes:ethyl acetate] (95:5, by vol.) and revealed by spraying with cupric sulfate and heating. MAMEs, mycolic acid methyl esters; FAMEs, fatty acid methyl esters. See Methods for details.
