# Supplementary material for: Cytokinin Signaling in Mycobacterium tuberculosis
Source: mBio. 2018 Jun 19;9(3):e00989-18. doi: 10.1128/mBio.00989-18 (PMC6016246; doi:10.1128/mBio.00989-18)
Supplement: FIG S5 [file mbo003183940sf5.pdf]

Samanovic, Hsu et al, Fig. S5

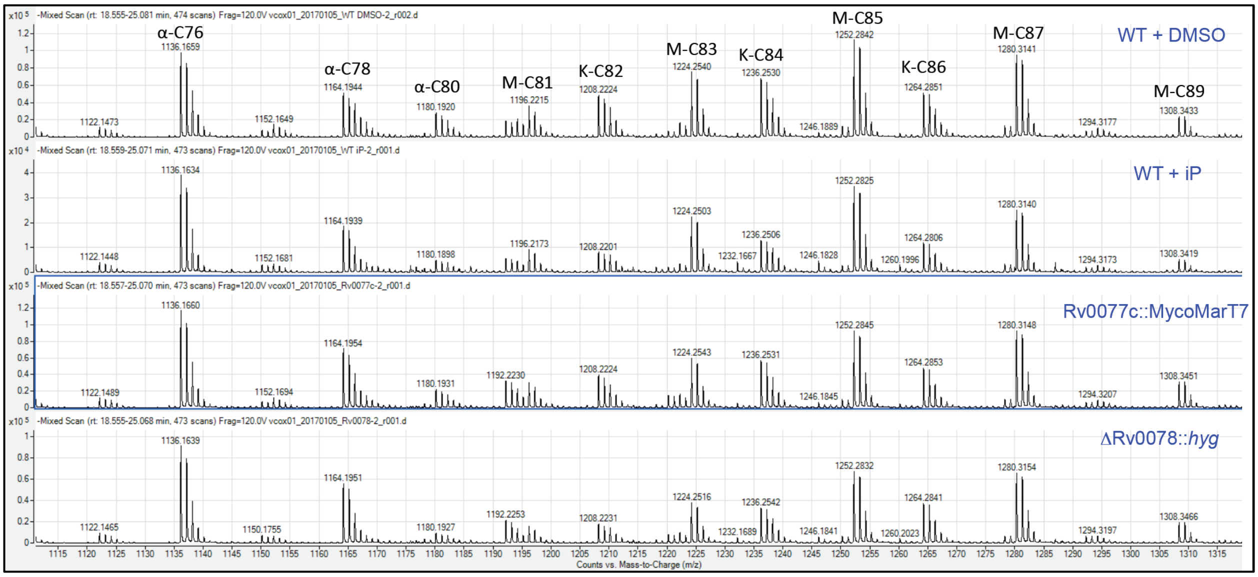

Fig. S5: Mix scan of the mycolic acid region (18.6 to 25 min) of the LC/MS runs for the wild-type *M. tuberculosis* either treated with the DMSO carrier alone or with the iP compound, the *Rv0077c* transposon mutant and the *Rv0078* allelic exchange mutant. The average mass spectrum across the elution times of all three classes of mycolates (alpha, α; methoxy, M; and keto, K) is presented for all four samples. The number of carbon atoms in each mycolic acid species is indicated above the corresponding peak. All four samples display comparable mycolic acid contents.
