# Supplementary material for: Cytokinin Signaling in Mycobacterium tuberculosis
Source: mBio. 2018 Jun 19;9(3):e00989-18. doi: 10.1128/mBio.00989-18 (PMC6016246; doi:10.1128/mBio.00989-18)
Supplement: TABLE S3 [file mbo003183940st3.docx]

**Table S3: GC/MS analysis of fatty acids**

Fatty acid methyl esters were prepared from total extractable lipids as described under Materials and Methods and analyzed by GC/MS. The percent areas of the fatty acids to the total areas of the GC/MS chromatograms in each species are shown in the table below.

|  | WT | WT  iP-treated | Rv0077c:*:*  MycoMarT7 | ΔRv0078*::*  *hyg* |
| --- | --- | --- | --- | --- |
| Myristic acid methyl ester (C14:0) | 1.3 | 1.3 | 1.2 | 1.0 |
| Palmitoleic acid methyl ester (C16:1) | 2.5 | 3.0 | 2.7 | 2.6 |
| Palmitic acid methyl ester (C16:0) | 24.0 | 25.8 | 26.9 | 25.5 |
| Oleic acid methyl ester (C18:1) | 33.0 | 32.5 | 29.1 | 35.5 |
| Stearic acid methyl ester (C18:0) | 12.0 | 13.6 | 12.4 | 14.4 |
| Tuberculostearic acid methyl ester (C19:0) | 11.5 | 10.5 | 10.8 | 11.6 |
| Eicosanoic acid methyl ester (C20:0) | 2.6 | 2.7 | 2.8 | 1.9 |
| Behenic acid methyl ester (C22:0) | 1.0 | 1.0 | 1.1 | 0.6 |
| Tetracosanoic acid methyl ester (C24:0) | 2.5 | 2.1 | 2.6 | 1.3 |
| Hexacosanoic acid methyl ester (C26:0) | 7.9 | 6.5 | 9.0 | 4.9 |
| Octacosanoic acid methyl ester (C28:0) | 1.7 | 1.0 | 1.4 | 0.7 |
